# Notes and Notices

**Published:** 1895-01

**Authors:** 


### NOTES AND NOTICES.

PRESIDENT MCCLELLAND, of the American Institute of Homœopathy, has appointed W. Tod Helmuth, M. D., a member of the Hahnemann Monument Committee, to fill the vacancy created by the decease of J. P. Dake, M. D.

REMOVAL NOTICE.—The offices of the Eclectic Medical Institute, *The Eclectic Medical Journal*, and John M. Scudder's Sons, Medical Publishers, have been changed from No. 228 West Court Street, to 301 Plum Street, above Court, Cincinnati, Ohio. P. O. Box 115.

### THE REAL VALUE OF THE MEDICINAL PEROXIDE OF HYDROGEN PREPARATIONS FOUND IN THE MARKET. By H. Endemann, Ph. D., Chemist, formerly Associate Chemist to the New York City Board of Health.

My attention having repeatedly been called to several reports and analyses made by different chemists and published by some medical journals, I concluded to examine all the brands of Peroxide of Hydrogen which I could find on the market, in order to ascertain the real value of each when intended to be used as an antiseptic remedy, both internally and externally.

The reports on the subject which have come to my knowledge are quite contradictory, and my object is to impart to the medical profession the results of my experiments, which have been made on fourteen fresh samples, purchased by me in duplicate directly from the manufacturers or their selling agents.

These brands have been tested for the volume of available oxygen, the amount of residue, the degree of acidity, and the amount of soluble Baryta Salts contained therein, as per following table:

| BRANDS OF $H_2O_2$ SOLUTION. | Volume of Available Oxygen, determined by means of a solution containing 5.665 Grammes of Permanganate of Potash per liter of distilled water. | Residue obtained from 100 c. c. of Hydrogen dried at 120° C. | Acidity expressed in cubic centimetres of Normal Volumetric Soda Solution for 100 c. c. of Peroxide. | Baryta found in Soluble Baryta Salts contained in 100 c. c. of Peroxide. |
|---|---|---|---|---|
| No. 1. Bene's $H_2O_2$ Medicinal, . . . . . . . . . . . . . . | 10.50 | 0.1886 | 2.19 | None |
| No. 2. Hydrozone, . . . . . . . . . . . . . . . . . . . . . | 27.35 | 0.2180 | 3.11 | " |
| No. 3. Larkin & Schefler's $H_2O_2$ Medicinal, . . . . . . . | 9.65 | 0.1206 | 6.75 | " |
| No. 4. Mallinckrodt's $H_2O_2$ Medicinal, . . . . . . . . . | 9.55 | 0.1408 | 1.43 | " |
| No. 5. Marchand's $H_2O_2$ Medicinal, . . . . . . . . . . . | 16.55 | 0.564 | 1.29 | " |
| No. 6. McKesson & Robbins' $H_2O_2$ Medicinal, . . . . . | 10.95 | 0.0540 | 0.44 | " |
| No. 7. Merck & Co.'s $H_2O_2$ Medicinal, . . . . . . . . . | 0.50 | 0.2418 | 4.57 | " |
| No. 8. Oakland Chemical Co.'s $H_2O_2$ Medicinal, . . . . | 10.50 | 0.0382 | 0.34 | 0.0017 |
| No. 9. Peuchot's $H_2O_2$ Medicinal, . . . . . . . . . . . . | 10.60 | 0.4674 | 1.77 | 0.0018 |
| No. 10. Powers & Weightman's $H_2O_2$ Medicinal, . . . . . | 8.40 | 0.0830 | 2.03 | None |
| No. 11. Pyrozone, 3 per cent., . . . . . . . . . . . . . . | 11.20 | 0.0534 | 0.76 | " |
| No. 12. Rosengarten & Son's $H_2O_2$ Medicinal, . . . . . . | 3.10 | 0.1002 | 0.25 | " |
| No. 13. Smith, Kline & French Co.'s $H_2O_2$ Medicinal, . . | 6.15 | 0.0880 | 2.6 | " |
| No. 14. E. R. Squibb's $H_2O_2$ Medicinal, . . . . . . . . . | 12.40 | 1.004 | 12.04 | " |

By referring to this table it is easily understood that sample No. 2, "Hydrozone," is far superior to any other brand which I have ever examined, not only on account of its containing a much larger amount of available oxygen, but also owing to the presence of a small quantity of several essential oils, the respective nature of which could not be determined, very likely because they have been submitted to the oxidizing action of Peroxide of Hydrogen before being used to make "Hydrozone."

I attribute to this small quantity of essential oils the great superiority of Hydrozone over any other brands of $H_2O_2$ as a healing agent.

When Hydrozone is diluted with distilled water, in the proportion of half and half, the resulting mixture contains about 13.5 volumes of available Oxygen, and its bactericide power still remains the same as the bactericide power of sample No. 5, which contains 16.55 volumes of available Oxygen.

Sample No. 14 comes next to sample No. 5, but it is readily seen that the degree of acidity is entirely too large for a preparation which is to be applied to the most sensitive diseased mucous membranes.

Sample No. 11, called "Pyrozone," which contains 11.20 volumes of available Oxygen, is quite similar to sample No. 6, with the exception that the latter contains a small quantity of Salicylic Acid. Very likely the Salicylic Acid has for its object to increase the bactericide power, but, unfortunately, I fear that it impairs the keeping properties of this preparation.

*Acidity.*—The fourteen brands which I have examined contain free acids (Phosphoric, Sulphuric, Muriatic); and I must say that Peroxide of Hydrogen Medicinal should never be made neutral before using, even in the most delicate cases. Neutral Peroxide of Hydrogen rapidly decomposes under all conditions of exposure.

The keeping properties of $H_2O_2$ solutions vary a great deal with the degree of purity and the percentage of free acids contained therein.

If the proportion of acid is too large, the profession well know that it acts as an irritant upon diseased surfaces. If it is too small, the solution don't keep well.

My opinion is that a standard solution of medicinal $H_2O_2$ must answer the following tests:

1. It should contain at least 15 volumes of available oxygen.
2. The quantity of free acids contained in 100 cubic centimetres should require not less than 1 c. c. and not more than 3 c. c. of normal volumeric Soda Solution, to be made neutral. Such a small quantity of free acid is not objectionable.
3. It should not contain any soluble Baryta Salts.
4. It must be free from sediment.

It is to be noticed that the brands No. 7 and No. 12 are valueless.

The brands No. 8 and No. 9 are not fit for medicinal uses, owing to the fact that they contain traces of soluble Baryta Salts.

The brand No. 3 has a heavy sediment of Sulphate of Baryta, which may be considered inert toward the system, but it is certainly detrimental to the keeping qualities of this preparation.

Brand No. 14, which is sold as a ten-volume solution, is really twelve volumes, but it is too acid. Brand No. 4, which is sold as a fifteen-volume solution, is really 16.55 volumes, viz.: About ten per cent. above the standard.

The brand No. 2, which is sold without any mention of volume, is really a 27.35 volume solution, viz.: Ninety per cent. above the standard.

None of the other brands come up to the standard, but, on the contrary, they run from thirty-five to fifty-five per cent. below.

—25 William Street, New York City.

### FUN FOR DOCTORS.

Roast Pig.—For seventy thousand years, according to wise Elia, man ate his pig meat raw. Then it befell that a man's cottage burnt down and roasted a pig. The people ate of the roasted pig and liked it better than the raw pig. So it became the fashion when a man wanted a feast to burn his house with a pig in it. Then one day a sage discovered that pigs could be roasted without burning the house. Since that time man has made no further advance in roasting pork. All of which goes to prove that human advance is a rather slow process, going by long-parted jumps, few in number.

For many cycles man believed it necessary to drain away his blood and burn out his mansion of clay with drugs in order to roast out the disease-devil that lurked within. One day the sage Hahnemann discovered that it was not necessary to burn the house in order to roast the disease. A good many wise people have taken that jump, but the majority of the world still hold to the old methods. Which, as remarked before, shows that advance is a very slow process—always excepting schemes and machines for getting money.

Heroic Homœopathy.—"How did you come to break both arms?" asked the sympathetic woman.

"It was Homœopathy did it, ma'am," replied the tramp. "I fell down and broke my right arm, and the doctor was one o' them similia smilibus fellers, so he broke the other."

Wrong Diagnosis.—Tired Harry.—"Lady, could yer help a poor feller a little? I've got a hackin' cough an' a headache."

Mrs. Kindlings.—"Well, I've got a little wood outside you could hack, and it might cure your headache."

Tired Harry.—"Much obleeged, mum; but, yer see, my headache ain't of ther splittin' kind."—*Puck.*

Safe from Ordinary Maladies.—"You'd better go away. We've got the measles here," said the woman at the kitchen door.

"Madam," replied the tramp, seating himself on the step with great deliberation, "the only disease I am afraid of is appendicitis. I shall be obliged to ask you, madam," he added, with dignity, "not to give me any cherry pie."—*Chicago Tribune.*

A Boy's Essay on Breath.—"Our breath is made of air. If it were not for our breath we would die. The breath keeps going through our liver, our lights, and our lungs. Boys shut up in a room all day should not breathe, they should wait until they get out of doors. Air in a room has carbonocide in it, and carbonocide is poisoner than mad dogs. Once some men was shut up in a black hole in India, a carbonocide got into that there hole and afore morning nearly every one was dead. Girls wear corsets which squeeze their diagrams too much. Girls cannot run and holler like boys, 'cause their diagrams are squeezed. If I was a girl I would just run and holler so my diagram would grow. That's all on breath."—*Doctor's Weekly.*

